# Pulsed lavage is associated with better quality of bone–cement–implant interface in knee arthroplasties (TKA/UKA) compared to syringe lavage in vitro; however, clinical data are missing: A systematic review

**DOI:** 10.1002/jeo2.12027

**Published:** 2024-05-20

**Authors:** Fabrizio Di Maria, Saubhik Das, Elisabeth Abermann, Christian Hoser, Christian Fink

**Affiliations:** ^1^ Gelenkpunkt‐Sports‐and Joint Surgery FIFA Medical Centre of Excellence Innsbruck Austria; ^2^ Research Unit for Orthopaedic Sports Medicine and Injury Prevention (OSMI), Medical Informatics and Technology Private University for Health Sciences Hall Austria

**Keywords:** jet lavage, knee arthroplasty, pulsed lavage, syringe lavage, TKA, UKA

## Abstract

**Purpose:**

The purpose of this systematic review is to analyse the available literature to ascertain the optimal method of bone preparation to improve the quality of bone–cement–implant interface with either pulsed lavage or syringe lavage in both total knee arthroplasty (TKA) and unicompartmental knee arthroplasty (UKA).

**Methods:**

A comprehensive search was conducted across MEDLINE, Scopus and Embase databases until July 2023. Both inclusion and exclusion criteria were clearly stated and used to identify all the published studies. Subsequent screening throughout the title, abstract and full text was made, followed by complete critical appraisal and data extraction. This sequential process was performed by two reviewers independently and summarised following the PRISMA (Preferred Reporting Items for Systematic Reviews and Meta‐Analyses guidelines). A quality assessment of the systematic review was performed according to the Quality Appraisal for Cadaveric Studies scale (QUACS), reaching a quality level ranging from 69% to 85%.

**Results:**

A total of 10 articles, out of 47, nine biomechanical cadaveric studies and one human clinical study were analysed. A total of 196 UKA tibial components, 74 patellar components, 36 TKA tibial components and 24 UKA femoral components were retrieved, and a high level of heterogeneity resulted overall. The pulsed lavage group showed better cement penetration and higher pull‐out force than the syringe lavage group; a higher interface temperature was also found in the pulsed lavage group. No differences were found regarding tension ligament forces between the groups.

**Conclusion:**

Our systematic review suggests that pulsed lavage is superior to syringe lavage in terms of the quality of bone–cement–implant interface in knee arthroplasties (TKA/UKA). However, translation of these results from cadaveric studies to individual clinical settings may be hazardous; therefore, clinical in vivo prospective studies are highly needed.

**PROSPERO CRD:**

PROSPERO CRD number CRD42023432399

**Level of Evidence:**

Level III.

**Abbreviations**
BMD: bone mineral density
PRISMA: Preferred Reporting Items for Systematic Reviews and Meta‐Analyses
QUACS: Quality Appraisal for Cadaveric Studies scale
TKA: total knee arthroplasty
UKA: unicompartmental knee arthroplasty

## INTRODUCTION

Both total knee arthroplasty (TKA) and unicompartmental knee arthroplasty (UKA) are able to alleviate pain and improve the function of end‐stage knee arthrosis with a reported rate of duration of more than 90% at 10–15 years [1, 20]. Despite knee prosthetic and surgical technique advancement, a sizeable number of patients require revision for various reasons; aseptic loosening of prostheses is reportedly one of the main reasons for the failure of the first implant [13, 24, 30]. A strong bone–cement–implant interface by withstanding tensile and shear forces is important for long duration and good outcomes [7, 31]. The strength of the interface relies on the amount of cement penetration into the resected bone surface and interdigitation with cancellous bone [19, 22, 25, 31, 33].

Among the multitude of factors that influence cement penetration, preparation and cleaning of bone surface before cementation with saline lavage to free it from bone debris, fat, bone marrow and blood is a crucial step, which can be accomplished with pulsed lavage or a syringe lavage—the two most frequently employed methods. Modern cementing technique advocates the use of pulsed lavage, the benefit of which in achieving satisfactory outcomes has been reported for both hip and knee arthroplasty [2, 9, 14, 25]. Clinical studies of knee arthroplasty comparing methods or giving details of bone bed preparation are lacking. Conceivably, there is still no consensus on the optimal method of bone surface preparation, neither is there any evidence to demonstrate which lavage technique gives a salubrious bone bed for optimal cement penetration and interlocking with cancellous bone. Although high‐impact pressure and high‐flow pulsatile lavage was demonstrated to improve cement penetration and stability of components in an experimental study [15], it remains to be seen whether it offers additional advantages compared to traditional syringe lavage, which would justify the additional cost for routinely using pulsed lavage for knee arthroplasty in highly competitive health care environment. This conundrum is evident in the fact that data from a survey shows the prevalence of pulsed lavage use not exceeding 70% for lower limb arthroplasties [17].

On this framework, we systematically reviewed current literature to answer whether pulsed lavage is better than syringe lavage in achieving greater cement penetration and interface stability in both TKA and UKA.

## MATERIALS AND METHODS

A systematic review of the literature was undertaken to identify studies comparing pulsed lavage and syringe lavage in TKA or UKA. The PRISMA (Preferred Reporting Items for Systematic Reviews and Meta‐analyses) guidelines were used to summarise the pathway for the selection of articles [21].

The review was registered on the PROSPERO database (No.: CRD42023432399). The research was performed by two independent investigators (F. D. M. and S. D.) using the MEDLINE, Scopus and Embase databases.

The following search string “arthroplasty, replacement, knee”[MeSH Terms] AND ((“puls*”[All Fields] AND “lavage”[Title/Abstract]) OR (“puls*“[All Fields] AND “irrigation”[Title/Abstract]) OR (“syringe lavage”[Title/Abstract] OR “syringe irrigation”[Title/Abstract]))” was used for MEDLINE. The same relevant terms were combined with Boolean operators for searching in other databases.

The following inclusion criteria were used for screening articles: (a) studies of any level of evidence; (b) studies written in English; (c) both clinical and cadaveric studies; (d) comparison between pulsed lavage and syringe lavage; (e) TKA or UKA. The exclusion criteria were as follows: (a) review articles, (b) case reports and (c) articles written in other languages. Two literature reviewers (F. D. M. and S. D.) independently screened all retrieved articles by title, abstract and full text in accordance with the defined inclusion and exclusion criteria. We excluded all the remaining duplicates, articles dealing with other topics, and those with poor scientific methodology or without an accessible full text. Reference lists were also hand‐searched for further identification of potentially relevant studies. Issues of disagreement were solved by a third author (E. A.).

Data from the included articles (type of study, patient demographics, methods, outcome measures and results) were extracted in Excel format and verified by a third author (E. A.).

## QUALITY ASSESSMENT

Since all except one of the studies are biomechanical cadaveric studies, a quality assessment of these articles was performed according to the Quality Appraisal for Cadaveric Studies scale (QUACS) [35].

The QUACS consists of a checklist encompassing 13 items. Each is to be scored with either 0 (*no/not stated*) or 1 (*yes/present*) points. Points are only assigned if a criterion is met without any doubt. The maximum score is 13. To enhance the comparability of results, a quality rating is expressed as a percentage value [reached score/maximum score (%)].

The assessments were performed by two authors (F. D. M. and S. D.) independently. Any discrepancy was discussed with the senior investigator for the final decision. All investigators agreed on the result of every stage of the assessment. The rating for each item is shown in Table S1. The quality range (minimum–maximum) was 69%–85%.

## RESULTS

From the search of MEDLINE, Scopus and Embase databases, 47 articles were screened, and following the inclusion and exclusion criteria, a total of 10 papers were considered eligible [3, 4, 8, 11, 12, 26, 27, 28, 29, 34].

**Figure 1:**
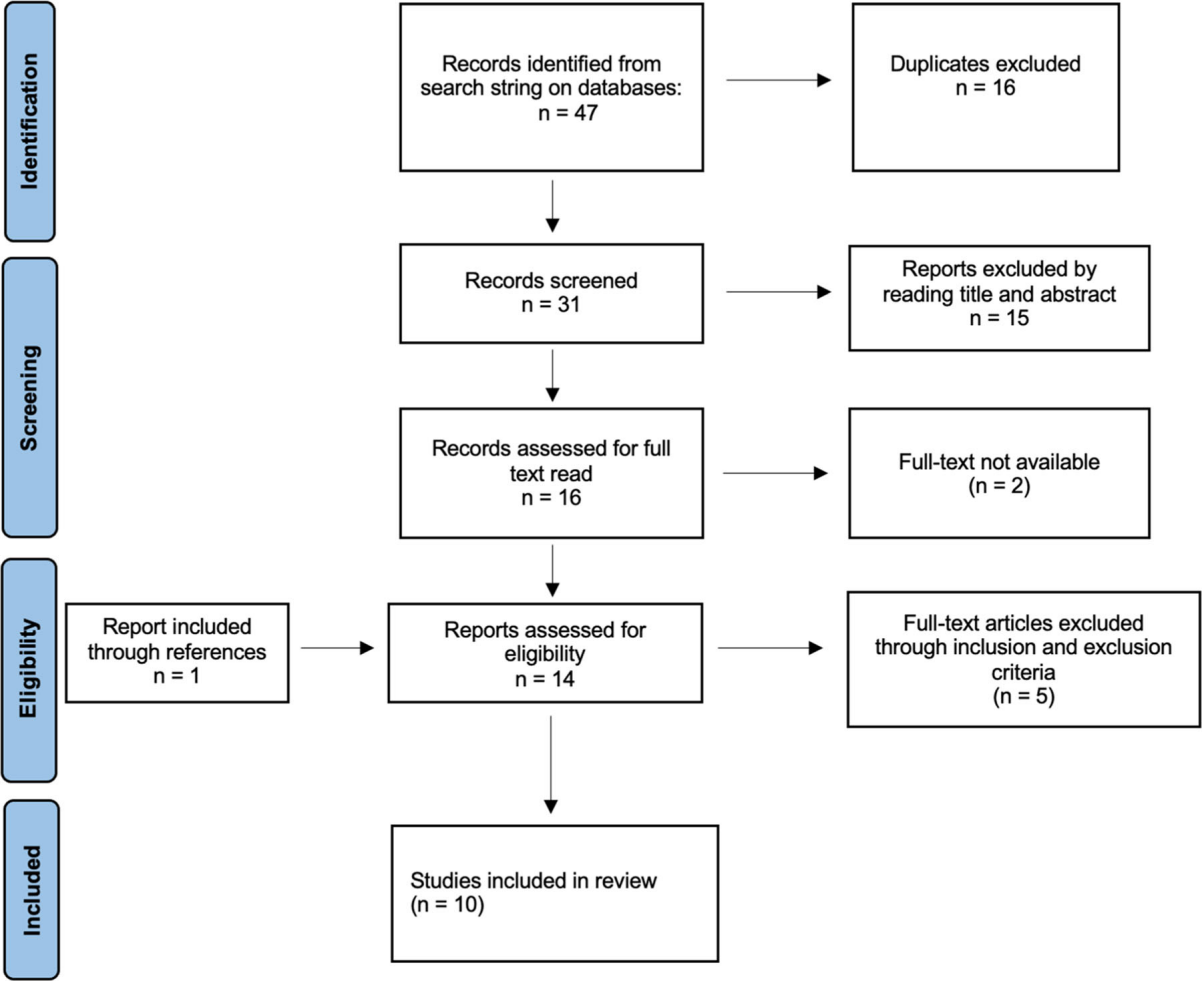
PRISMA (Preferred Reporting Items for Systematic Reviews and Meta‐Analysis) flowchart of the systematic search.

A PRISMA flowchart [21] of the method of screening and selection method is provided (Figure 1).

All the articles selected for the present review, except one, were biomechanical cadaveric studies. Five studies evaluated outcomes on the UKA tibial component, whereas two dealt with the TKA tibial component, one with the UKA femoral component, one on patellar polyethylene, and one with the experimental polyethylene tibial component. A total of 112 UKA human tibiae, 84 UKA cadaveric tibiae, 74 cadaveric patellae, 36 TKA cadaveric tibiae and 24 UKA cadaveric femoris were included in the study and the bone–cement–implant interface was analysed.

Overall, data from the selected studies were rather heterogeneous, and a summary of the principal characteristics is provided in Table 1.

**Table 1:** Summary of main characteristics of all included studies.

Authors	Year	Type of article	Sample	Methods	Outcome measure	Results
Clarius et al.	2009	Case–control study Human patients	Group A (pulsed lavage) *n* = 56 27 M, 27 F Mean age = 63 Group B (syringe lavage) *n* = 56 24 M, 22 F Mean age = 68	Oxford UKA Same technique, different surgeon, different cement Same postop protocol Comparison between Group A and B	X‐ray FU Minimum FU = 12 months RLs Cement penetration (mm)	Overall incidence of RL = 58% No pathological RL in either group Less incidence of RL in group A in the medial portion of bone–cement interface (*p* < 0.05) Better cement penetration in Group A (*p* < 0.05)
Clarius et al.	2012	Case–control study Cadaveric study	24 paired fresh‐frozen legs Mean age = 63.5 years Group A (pulsed lavage) *n* = 12 Group B (syringe lavage) *n* = 12	Oxford UKA tibial components Same technique, same cement Comparison between Group A and B	Bone–cement interface temperature (Celsius degree) Cement penetration area (mm^2^) Ligament tension force (N)	Higher interface temperature in Group A Higher cement penetration area in Group A No statistical difference in ligament tension force
Jaeger et al.	2013	Case–control study Cadaveric study	20 paired fresh‐frozen legs Group A (pulsed lavage) *n* = 10 Group B (syringe lavage) *n* = 10	Oxford UKA tibial components Same technique, same cement Comparison between Group A and B	Cement penetration (mm) Cement volume under the tibial tray	Higher cement penetration in Group A Higher cement volume under the implant in Group A
Jaeger et al.	2014	Case–control study Cadaveric study	22 paired fresh‐frozen legs Mean age = 74.4 years Group A (pulsed lavage) *n* = 11 Group B (syringe lavage) *n* = 11	Oxford UKA tibial components Same technique, same cement Comparison between Group A and B	Cement penetration (mm) Micromotion and subsidence	Higher cement penetration in Group A Group A showed reduced both micromotion and subsidence (*p* < 0.05)
Helwig et al.	2013	Case–control study Cadaveric study	12 fresh‐frozen tibial plateau specimens Group A (pulsed lavage) *n* = 3 Group B (syringe lavage) *n* = 3 Group C (brush cleaning) *n* = 3 Group D (no cleaning) *n* = 3	Experimental polyethylene tibial plate Same technique, same cement Comparison between Group A, B, C and D	Cement penetration (mm)	Higher cement penetration in Group A, B and C compared to Group D. Intragroup difference showed that Group A had higher cement penetration than the others. No difference was found between Group B and C
Scheele et al.	2016	Case–control study Cadaveric study	18 fresh‐frozen tibial plateau specimens Mean age = 72.2 years Group A (pulsed lavage) *n* = 6 Group B (syringe lavage) *n* = 6 Group C (brush cleaning) *n* = 6	Univation (Aesculap) UKA tibial component Same technique, same cement Comparison between Group A, B and C	Cement penetration (mm) Load failure	No difference in cement penetration and load failure between groups
Schlegel et al.	2011	Case–control study Cadaveric study	12 fresh‐frozen tibial plateau specimens Mean age = 71.5 years Group A (pulsed lavage) *n* = 6 Group B (syringe lavage) *n* = 6	PFC Sigma Knee System (DePuy) TKA tibial component Same technique, same cement Comparison between Group A and B	Cement penetration (mm) Pull‐out force (N)	Higher cement penetration in Group A Higher pull‐out force in Group A
Schlegel et al.	2014	Case–control study Cadaveric Study	12 fresh‐frozen tibial plateau specimens Mean age = 71 years Group A (pulsed lavage with finger packing of cement) *n* = 6 Group B (syringe lavage with gun cementation) *n* = 6	PFC Sigma Knee System (DePuy) TKA tibial component Different technique (finger packing vs. gun cementation), same cement Comparison between Group A and B	Cement penetration (mm) Pull‐out force (N)	Higher cement penetration in Group A Higher pull‐out force in Group A
Seeger et al.	2013	Case–control study Cadaveric study	24 paired fresh‐frozen legs Group A (pulsed lavage) *n* = 12 Group B (syringe lavage) *n* = 12	Oxford UKA Femoral Component Same technique, same cement Comparison between Group A and B	Cement penetration area (mm^2^) Bone–cement interface temperature (Celsius degree) Ligament tension force (N)	Higher cement penetration area in Group A Higher interface temperature in Group A No difference in ligament tension force
Weiss et al.	2003	Case–control study Cadaveric study	74 paired fresh‐frozen human cadaver patellae Group A (pulsed lavage) *n* = 37 Group B (syringe lavage) *n* = 37	PFC Sigma (Johnson and Johnson) patellar component Same technique, same cement Comparison between Group A and B	Cement penetration (%) in 24 specimens Mean pull‐out force (N) in 50 specimens	Significantly greater cement penetration in Group A Significantly higher pull‐out force in Group A

Abbreviations: F, female; FU, follow‐up; M, male; RL, radiolucent lines; TKA, total knee arthroplasty; UKA, unicompartmental knee arthroplasty.

The major outcome analysed was bone–cement penetration in all studies.

**Figure 2:**
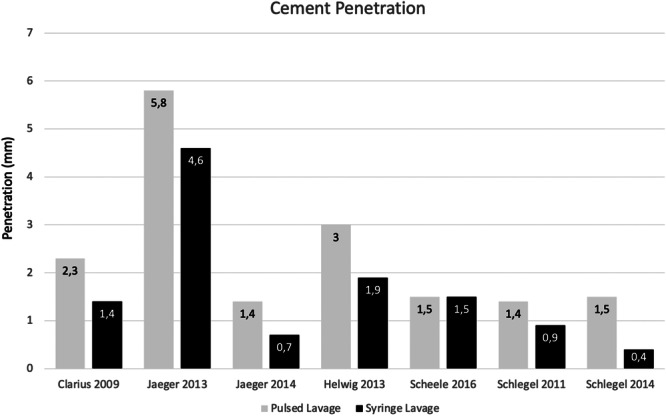
Mean cement penetration (mm) in pulsed and syringe lavage groups. A statistical difference was found in all studies except Scheele et al. [26].

This is shown to be superior in the pulsed lavage group in all studies except Scheele et al. [26], where no statistical significance was found. A summary graph of cement penetration (mm) can be found in Figure 2. Three studies were not included in the graph due to the heterogeneity of measurements, as they expressed values in percentage or mm^2^ [4, 29, 34].

**Figure 3:**
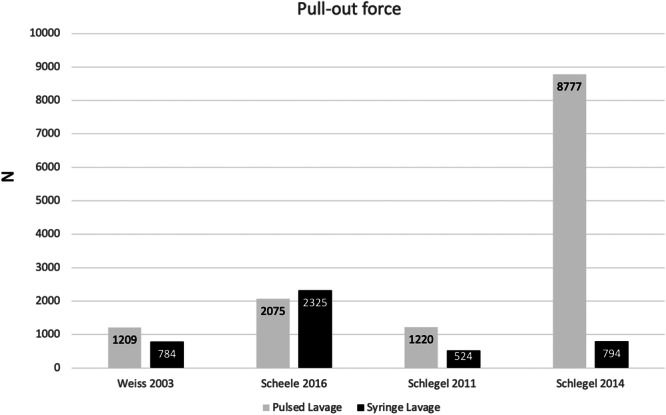
Mean pull‐out force (N) in pulsed and syringe lavage groups. A statistical difference was found in all studies except Scheele et al. [26].

Secondary, pull‐out force was recorded in four studies. A summary graph is shown in Figure 3. Only Scheele et al. found a higher pull‐out force in the syringe lavage group [26], without statistical significance between groups (*p* = 0.910), whereas the others found statistically higher values to pull out the implant in the pulsed lavage group.

**Figure 4:**
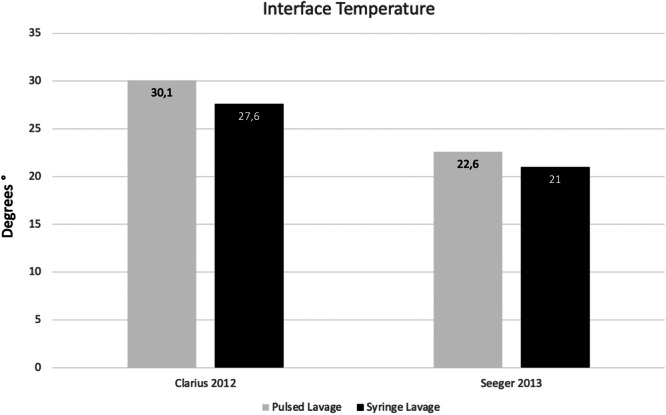
Mean interface temperature (Celsius degrees) in pulsed and syringe lavage groups. A statistical difference was found in both studies.

Regarding interface temperature, two studies showed significantly higher temperatures in the pulsed lavage group [4, 29]. A summary graph is shown in Figure 4. Although the absolute value differs among the studies, the difference between groups is very similar. Therefore, this may be due to the inherent sensitivity of the thermometer.

Lastly, two studies evaluated ligament tension force between groups, and no statistical differences were found [4, 29].

Only Clarius et al., in their human case–control study, assessed the prevalence in radiolucent lines with 12 months or more postop x‐rays [3]. They found less prevalence on radiolucent lines in the pulsed lavage group. In spite of that, neither group showed pathological radiolucent lines using the threshold value of 2 mm.

## DISCUSSION

The burgeoning number of knee arthroplasties is paralleled by a growing interest in the advancement of surgical procedures to prolong the longevity of prostheses and improve outcomes. Albeit novel cementation techniques along with different manoeuvres for resected bone surface preparation are now available, concerns remain about consensus for optimal bone surface preparation [2, 25].

Accurate bone lavage is a prerequisite before cementation, and pulsed lavage or syringe lavage are the most common procedures. However, evidence of the superiority of one method over the other is lacking so far. In this systematic review, we found that pulsed lavage, compared to syringe lavage, is associated with greater cement penetration and high interface stability of prostheses in both TKA and UKA.

Achieving optimal cement penetration is of pivotal importance to ensure satisfactory bony interdigitation culminating in a strong prosthetic bone–cement interface [19, 25, 31, 33]. There is still ambiguity with regard to what constitutes optimal cement penetration, but it is generally agreed that 3–5 mm of penetration depth provides adequate stability, while penetration of 1.5 mm or less is associated with aseptic radiolucency and risk of subsequent loosening [15, 25, 31].

Our systematic review has shown that bone surface cleaning with pulsed lavage resulted in significantly greater cement penetration depth under the tibial component compared to syringe lavage [3, 8, 11, 12, 27, 28]; likewise, significantly greater cement penetration area was also reported when pulsed lavage was used for preparation [4, 12].

One cadaveric experimental study assessed femoral cement penetration in UKA after pulsed lavage or syringe lavage and showed that cancellous bone preparation with pulsed lavage led to a significantly higher cement penetration area [29]. Significantly greater cement penetration depth was also obtained under polyethylene patellar component after preparing with pulsed lavage [34]. Scheele et al., however, evaluated cement penetration under the tibia component in a study of 18 human cadaver knees implanted with UKA and compared pulsed lavage, brush cleaning and control. Although they did not find any significant difference in the depth of cement penetration between groups, pulsed lavage and brush preparation significantly reduced the area without cement penetration compared to the control group. Furthermore, brush preparation favoured cement penetration in the crucial posterior part of the tibia more than in pulsed lavage [26]. In contrast, Helwig et al. showed superiority of pulsed lavage over other methods. They assessed cement penetration under the tibial component in human tibial plateau specimens and compared four different methods, namely, pulsed lavage, brush cleaning, syringe lavage and no cleaning. Results showed that pulsed lavage yielded a significantly greater depth of cement penetration and the longest bone–cement contact than any other method [8].

Cement penetration can also be influenced by the method of application; many advocate gun‐cementing rather than finger packing of the tibial surface to improve penetration [18, 25]. Schlegel et al., however, demonstrated that pulsed lavage with finger packing of cement achieved superior cement penetration and interface strength than syringe lavage combined with gun cementing [27]; therefore, pulsed lavage seems critical for cementation and mere gun cementing alone cannot surrogate the benefit of pulsed lavage.

Cement penetration is also dependent on pressurisation, where ligament tension plays a major role in knee arthroplasties to maintain continuous pressure [10]. Finally, no statistical difference between pulsed and syringe lavage was found in other studies [4, 29].

Greater cement penetration would ensure a stronger interdigitation with bone trabeculae thus leading to a durable bone–cement–implant construct to resist load and shearing forces [19, 22, 25, 33]. The present review also shows significantly higher median pull‐out forces required for mechanical failure of tibial components in TKA when pulsed lavage was used before cementation [27, 28]. However, Scheele et al. did not find any significant difference in the primary stability of the tibial component of UKA under dynamic compression‐shear test conditions when comparing between pulsed lavage, brush preparation and control group [26]. Weiss et al. demonstrated that the maximum pull‐out force for mechanical failure of the patellar component was significantly greater in pulsed lavage specimens compared to syringe lavage [34]. The mode of failure of the tibial component under pull‐out forces depends on the depth of cement penetration since tibial components mainly failed at the bone–cement interface in syringe lavage specimens indicating lower interface strength [27, 28]. The stability of prosthetic components also depends on bone quality since suboptimal bone mineral density (BMD) negatively impacts component subsidence and micromotion [36]. Jaeger et al. showed that pulsed lavage offset the effect of BMD; in fact, pulsed lavage specimens had significantly less tibial tray micromotion and subsidence than syringe lavage [11]. Deeper cement penetration combined with reduced implant micromotion would protect against radiolucency under components, which might be a precursor of aseptic loosening [11]. Beneficial effect of pulsed lavage is also evident in the observation of significantly lower incidence of radiolucent lines under tibial tray in a case–control study of 112 cemented UKAs [3].

With greater penetration of cement, there comes a potential risk of thermal damage and necrosis of the cancellous bone during cement polymerisation, which might lead to osteolysis and subsequent loosening of the component [6, 32]. Thermal injury to bone is both time and temperature‐dependent, with temperatures at or below 44°C reportedly not producing osseous injury, whereas temperatures of 47–50°C applied for more than 1 min resulting in bone reabsorption and vascular injury [5]. Seeger et al. observed significantly higher interface temperature in the femur in pulsed lavage specimens of UKAs; however, the maximum temperature was approximately 26°C, which is far below the critical level producing bone damage [29]. Clarius et al. also reported higher interface temperature in the tibia in the pulsed lavage group [4]; however, it remained below the critical threshold temperature and lasted for a shorter time to produce any substantial bone damage [5]. It is evident that with a greater amount of cement in cancellous bone, the interface temperature goes higher after cleaning with pulsed lavage; however, it has not yet been demonstrated to negatively affect the component stability and outcome. Thus, the benefit of greater cement penetration might outweigh the theoretical risk of thermal damage in knee arthroplasties [16].

This study has limitations. Except for one, all the included studies are experimental cadaveric studies, which have their own inherent shortcomings. The assessment of cement penetration in the studies was not uniform. Results from cadaveric studies may differ from actual clinical intraoperative situations in view of bone quality, surrounding soft tissues and ligament tension and the presence of blood.

The substantial heterogeneity of studies, consisting of inclusion of both TKA and UKA, measurements made on either tibia or femur or patella, report of depth or area/volume of penetration and the different types of used cement, makes meta‐analysis unsuitable and prevents achieving firm conclusions.

Furthermore, the testing condition for interface stability assessment is a simplification of complex dynamic forces around the knee in a real clinical situation. The results from this systematic review need to be interpreted with caution. However, to the best of our knowledge, this study is purportedly the first one to systematically investigate this clinically relevant issue with all the evidence available so far. Nonetheless, it provides us with an insight that might be important for further clinical studies. We believe that these are strongly needed and performed according to reproducible methods based on stereophotogrammetric analysis to evaluate micromovements of prosthetic components and their correlation to prosthesis duration [23].

## CONCLUSION

This review shows that variability in findings among studies is often present. In addition, almost all of the studies in this review are cadaveric, which makes it very difficult to transfer these results to the clinical setting. Although pulsed lavage has shown better results and seems to generate a better bone–cement interface, and component stability without any adverse consequences under these circumstances, the reporting of purported advantages of pulsed lavage over syringe lavage cannot be meant to create a dogma.

## AUTHOR CONTRIBUTIONS

**Fabrizio Di Maria**: Conceptualisation; methodology; data curation and synthesis; writing—original draft preparation; writing—review and editing. **Saubhik Das**: Conceptualisation; methodology; data curation and synthesis; writing—original draft preparation; writing—review and editing. **Elisabeth Abermann**: Conceptualisation; writing—original draft preparation; writing—review and editing. **Christian Hoser**: Conceptualisation; data curation and synthesis; writing—review and editing. **Christian Fink**: Conceptualisation; methodology; data curation and synthesis; writing—original draft preparation; writing—review and editing; supervision. All authors interpreted the data, critically reviewed the work, made important contributions to the manuscript with their title page suggestions for improvement, approved the published version and agreed to be responsible for all aspects of the work. All authors consent to the publication of the manuscript. All authors have read and agreed to the published version of the manuscript.

## CONFLICT OF INTEREST STATEMENT

The authors declare no conflict of interest.

## ETHICS STATEMENT

Not applicable.

## Supporting information

Supporting information.

## Data Availability

The data sets used and/or analysed during the current study are available from the corresponding author upon reasonable request.
