# Supplementary material for: Pulsed lavage is associated with better quality of bone–cement–implant interface in knee arthroplasties (TKA/UKA) compared to syringe lavage in vitro; however, clinical data are missing: A systematic review
Source: J Exp Orthop. 2024 May 20;11(3):e12027. doi: 10.1002/jeo2.12027 (PMC11106550; doi:10.1002/jeo2.12027)
Supplement: Supplementary file 1 — Supporting information. [file JEO2-11-e12027-s001.docx]

**Supplementary Materials**

**Table S1.** Quality assessment of the cadaveric studies using QUACS Scale [34] are presented. No/Not stated judgement (red circles) or Yes/Present judgement (green circles).

**
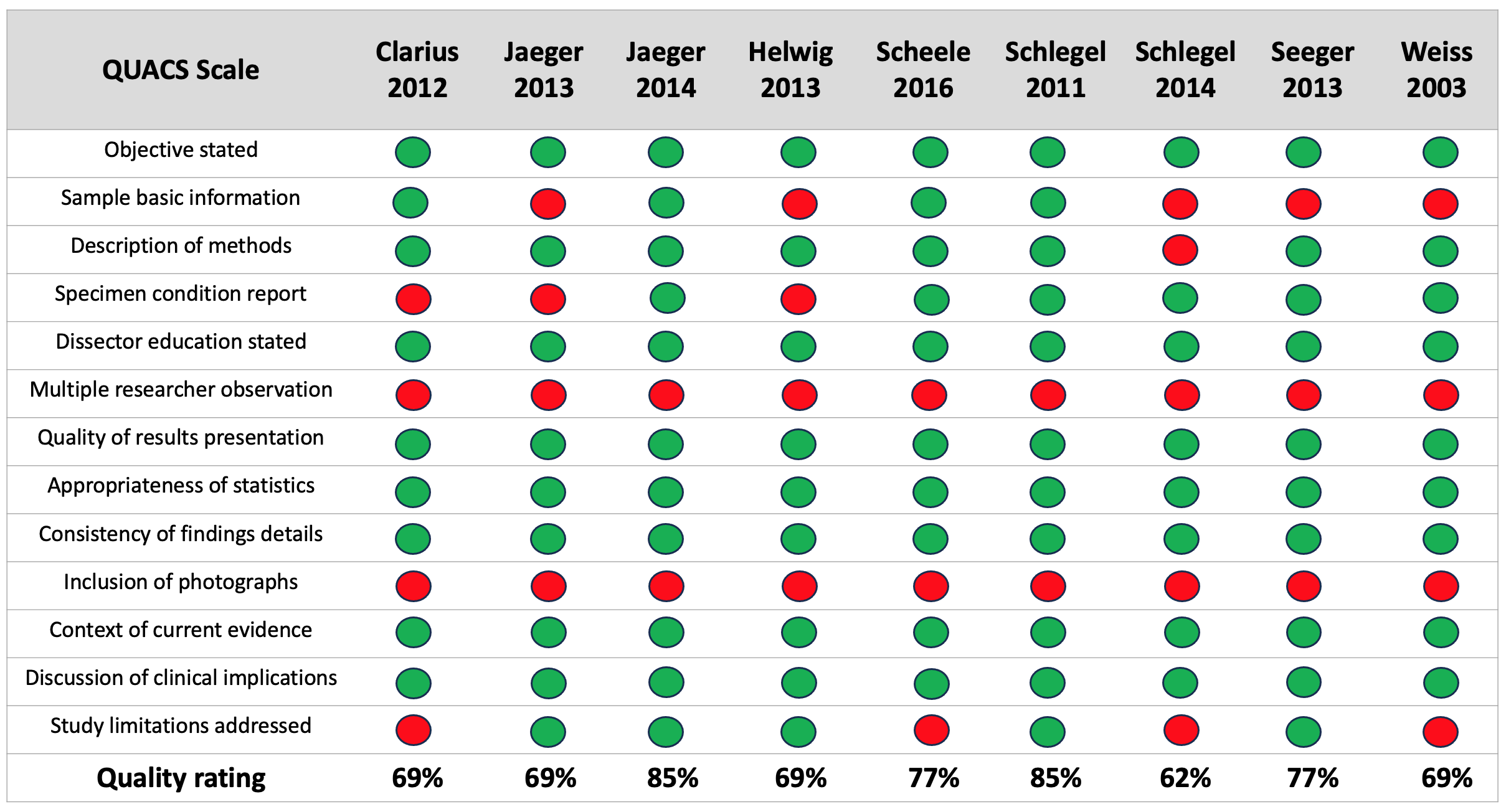
**
